# Centenarian Mortality Rate During COVID-19: Protocol for a Systematic Review and Meta-Analysis

**DOI:** 10.2196/74068

**Published:** 2025-08-13

**Authors:** Shaima Ibrahim, Omnia Mahmoud Abdelraheem, Wafa Abu El Kheir-Mataria, Sungsoo Chun

**Affiliations:** 1 Institute of Global Health and Human Ecology American University in Cairo New Cairo Egypt

**Keywords:** coronavirus, pandemic, mortality, COVID-19, older adults, centenarians, systematic review, meta-analysis

## Abstract

**Background:**

Marked by high mortality rates on a global scale, with disease mortality being notably focused among older adults, the COVID-19 pandemic has become a significant health crisis. Despite the numerous publications on COVID-19 mortality among older adults, there is still a gap in knowledge when considering centenarians, as there is no systematic review and meta-analysis that summarizes COVID-19 mortality in centenarians globally.

**Objective:**

This study aims to systematically review and synthesize global evidence on COVID-19 mortality rates among centenarians and the population of older adults worldwide, whether residing in long-term health facilities, hospitals, or their homes.

**Methods:**

An automated search was conducted on the following databases: PubMed, Scopus, and Web of Science. Observational studies, both cohort and case-control, were selected. Quality assessment of the selected studies was based on the Joanna Briggs Institute critical appraisal tool for observational cohort and case-control studies. Three independent authors conducted the searches, and any possible disagreements were resolved by consensus. A meta-analysis of mortality proportions will be conducted to calculate the raw, logit, and arcsine proportions for all studies included in our meta-analyses. Heterogeneity between studies with a significance of *P*=.05 will be assessed by calculating the *I*^2^ value using the DerSimonian and Laird method for random effects. Odds ratios and 95% CIs for dichotomous data and weighted mean risk differences and 95% CIs for continuous variables will be calculated. Further subgroup analyses will be attempted to explore heterogeneity among over 6.7 million older adults. Leave-one-out sensitivity tests will be conducted to assess the robustness of our results. The meta-analysis will be conducted using R software version 4.4.2 (R Foundation for Statistical Computing).

**Results:**

A total of 4 studies were included in our systematic review and meta-analysis. Of the included studies, 3 are retrospective cohort studies and 1 is an observational, retrospective case-control study. As for study group size, 1 cohort study was conducted on a population of less than 1000 participants, 2 studies (1 cohort and 1 case-control) involved more than 10,000 participants, and 1 cohort study included more than 6 million participants.

**Conclusions:**

This study has significant potential. Given the consensus that older adults, let alone centenarians, are the most vulnerable demographic to serious outcomes and deaths during pandemics. Addressing these gaps is crucial for the informed development of public policies, enabling countries to minimize the impacts on this population, particularly during health crises such as the COVID-19 pandemic.

**Trial Registration:**

PROSPERO CRD42025645150; https://www.crd.york.ac.uk/PROSPERO/view/CRD42025645150

**International Registered Report Identifier (IRRID):**

DERR1-10.2196/74068

## Introduction

### Overview

Novel coronavirus cases were first detected in China in December 2019, with the virus spreading rapidly to other countries worldwide. This led the World Health Organization to declare a public health emergency of international concern on January 30, 2020, and to mark the outbreak as a pandemic on March 11, 2020 [1,2]. The aim of this study is to conduct a systematic review and meta-analysis of studies published between December 2019 and December 2024 on the rate of COVID-19 mortality in centenarians (ie, individuals aged 100 years and older) versus older adults aged 60-99 years (hereafter referred to simply as other older adults) [3]. Since the beginning of the pandemic, more than 777 million people have contracted the severe acute respiratory syndrome coronavirus-2 (SARS-CoV-2) globally, and over 7.1 million people have lost their lives due to COVID-19 to date [4]. Mortality from COVID-19 increases with age, with children being the least susceptible to death [5,6]. Italy was the first European country to be affected by COVID-19 [7]. The biggest cluster of cases occurred in Lombardy, the most populous Italian region, and older adults were hit in the hardest way [8]. In this population, Marcon et al [8] questioned if the COVID-19 mortality in centenarians was lower than that in other older adults and whether sex differences exist in mortality among different age classes. Comparisons were made using total mortality (ie, not only confirmed COVID-19 cases) at the peak of infection (March 2020) against March’s total mortality of previous years. They did not find reduced mortality in centenarians relative to other older adults but highlighted a difference between sexes across different age classes. While mortality in those aged 60-99 years was much higher in men than in women, the rate at which the risk increased by age was slower in men than in women, such that centenarian women had a higher mortality rate. They suggested that the proinflammatory status of older adults, referred to as inflammageing, could explain such age-related vulnerability. Despite the observations of multiple studies measuring the mortality rate in older adults, studies concerning mortality in centenarian patients with COVID-19 remain very scarce [9,10]. Addressing this gap is essential to reinforce our understanding of the unique challenges faced by centenarians and enable more effective health planning. This, in turn, facilitates the development of targeted treatment approaches with proper interventions tailored to the specific health needs of this demographic, particularly in situations of health crises like the COVID-19 pandemic [11-13]. In view of the foregoing, the aim of this study is to investigate the mortality rates in centenarians worldwide due to COVID-19.

### Objective

The aim of this study is to conduct a systematic review and meta-analysis of studies published between December 2019 and December 2024 on the rate of COVID-19 mortality in centenarians versus other older adults.

## Methods

### Study Protocol and Meta-Analysis Strategy

The protocol for our systematic review and meta-analysis was conducted following PRISMA (Preferred Reporting Items for Systematic Reviews and Meta-Analyses) guidelines [14]. Our meta-analysis study will be conducted in compliance with the guidelines detailed in the Cochrane handbook for systematic reviews of interventions [15].

### Eligibility Criteria

#### Inclusion Criteria

**Figure 1:**
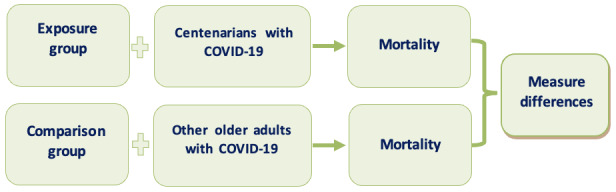
PICOS (population, intervention, comparison, outcomes, and study) design for eligibility criteria.

The PICOS (population, intervention, comparison, outcomes, and study) design for eligibility criteria [16] was adopted in this study (Figure 1). The population of interest will be individuals aged 100 years and older. The intervention will be testing positive for COVID-19. The comparison group will be individuals aged 60-99 years. The outcome of interest will be mortality rates in both populations from COVID-19. The studies included will comprise only peer-reviewed, longitudinal observational cohort and case-control studies published from December 2019 until December 2024 in English.

#### Exclusion Criteria

The following studies will be excluded from the meta-analysis: (1) studies that do not include centenarians, (2) mortality studies during the COVID-19 pandemic that include deaths not due to COVID-19, (3) studies that do not include individuals younger than 100 years, (4) studies that do not present mortality rates as their effect measure, and (5) studies that do not fit or address our research question. Systematic reviews, scoping reviews, and book records will also be excluded, as well as research papers not available in English. The restriction regarding publication time (December 2019 to December 2024) is meant to reflect the temporality of the COVID-19 pandemic and at the same time widen our search window to include studies that were published after the pandemic was declared over in May 2023 [17].

### Information Sources, Search Strategy, and Study Selection

The PubMed, Scopus, and Web of Science electronic search databases were consulted by 2 researchers on January 7, 2025, to search for studies published between December 2019 and December 2024 to identify any cohort and case-control studies that investigated the relationship between COVID-19 diagnosis and mortality in centenarians versus other older adults. The main keywords used were “centenarians” and “covid” in addition to their variations (Table 1). In the search strategy, keywords were systematically combined using the Boolean operators “AND” and “OR” to refine and expand the retrieval of relevant literature. The references of the studies included in the full-text evaluation phase were reviewed independently by the 2 researchers to identify potentially relevant studies that were not considered in earlier search phases. The studies were screened against the eligibility criteria in 2 phases: title and abstract screening followed by full-text screening. In cases of disagreement between the 2 reviewers at any stage, a consensus process was undertaken. If a resolution was not reached, a third reviewer was consulted for resolution. If data are missing or unclear, attempts will be made to contact the study authors for clarification. If contact cannot be established, the study will be excluded from our analysis, and this will be addressed in the discussion section. Science reviews, systematic reviews, and meta-analyses found in the automated search were excluded from our study.

The data to be extracted from the selected studies will include (1) author and year of publication, (2) name of the journal, (3) study design, (4) country of origin of the study, (5) study objective, (6) sample size, (7) period of data collection, (8) statistical test used, (9) age of participants, (10) sociodemographic details (ie, living alone or in a long-term health facility), (11) COVID-19 status (ie, positive or negative), and (12) measured outcome. A spreadsheet in Microsoft Excel will be used to record the necessary data for running the meta-analysis. Data will be presented in tables and charts, and their interpretation will be discussed.

**Table 1:** Search strategy.

Database	Query	Number of studies
PubMed	(“centenarians”[MeSH Terms] OR “centenarians”[All Fields] OR “centenarian”[All Fields]) AND (“sars cov 2”[MeSH Terms] OR “sars cov 2”[All Fields] OR “covid”[All Fields] OR “covid 19”[MeSH Terms] OR “covid 19”[All Fields])	34
Scopus	( TITLE-ABS-KEY ( centenarian AND covid ) ) OR ( TITLE-ABS-KEY ( *supercentenarian* OR semi*supercentenarian ) AND ORIG-LOAD-DATE AFT 20240314 ) AND ( LIMIT-TO ( DOCTYPE , “ar” ) OR LIMIT-TO ( DOCTYPE , “no” ) OR LIMIT-TO ( DOCTYPE , “cp” ) OR LIMIT-TO ( DOCTYPE , “sh” ) OR LIMIT-TO ( DOCTYPE , “le” ) OR LIMIT-TO ( DOCTYPE , “ed” ) )	29
Web of Science	(ALL=(centenarian)) AND ALL=(covid)	38

### Risk of Bias and Study Quality Assessment

Funnel plots will be structured to visually assess publication bias in our meta-analysis [18]. They will be structured as scatter plots, with study effect sizes on the x-axis and a measure of study precision (standard error) on the y-axis. Because visual inspection can be subjective, statistical Egger regression tests will supplement visual assessment for more robust conclusions. In addition, trim-and-fill analysis [19] will be considered to assess publication bias and display the heterogeneity of the studies included in the systematic review. The Joanna Briggs Institute critical appraisal tool will be used to evaluate the quality of observational cohort and case-control studies [20]. The quality assessment for the selected literature will be evaluated independently by all authors (Multimedia Appendix 1).

### Synthesis of Results

Our preliminary search in the 3 databases resulted in a total of 101 research papers. An additional paper was found in Google scholar, totaling 102 papers. A third researcher was consulted for help with screening the 102 articles, reviewing their respective abstracts and removing duplicates. This resulted in 54 articles remaining. The 54 articles were downloaded and fully reviewed to check their eligibility for our study. Data will be extracted from the chosen studies into tables and charts, and their interpretation will be discussed. Overall mortality proportions will be compared among studies to calculate pooled raw, logit, and arcsine proportions for all studies included in our meta-analysis. A random effects model for meta-analyses will be calculated using the DerSimonian and Laird method. This model acknowledges that studies included in the analysis may have different underlying effect sizes, rather than assuming a single true effect size across all studies, as in the fixed-effect model [21]. This means that, in addition to the within-study variability, there is also between-study heterogeneity that needs to be accounted for. The random effects model estimates both the within-study (*I*^2^) and between-study (τ²) variances. Heterogeneity will be evaluated with a significance level of *P*=.05. An assessment of heterogeneity with *I*^2^ values will be presented. Heterogeneity of around 25% will be considered low, around 50% moderate, and around 75% high. The arcsine transformation of proportions will be primarily considered in our meta-analyses because it stabilizes the variance of proportion data, especially when proportions are close to 0 or 1 (ie, when studies report very low or very high proportions and variance instability is most pronounced) [22]. Arcsine transformation makes the data more suitable for standard meta-analytic analysis techniques that assume normality and homogeneity of variance. Weighted mean risk differences and 95% CIs will be calculated for continuous variables. Odds ratios and 95% CIs for each dichotomous data outcome will also be determined [23]. Individual study results will be visually summarized using forest plots to display both individual study estimates and the pooled estimate from the meta-analysis [15]. Meta-analyses will be conducted using R software version 4.2.2 (R Foundation for Statistical Computing).

### Subgroup Analysis and Sensitivity Test

Further subgroup analyses will be conducted considering other factors such as country, age brackets, and long-term care facility versus community dwelling to explore the heterogeneity among the 6.7 million older adults included in our study [24]. This is to help explain whether there is a variation in effect sizes across studies. Leave-one-out sensitivity tests will illustrate how far the calculated pooled effect estimate shifts when each study is excluded one at a time and recalculating the pooled effect size [25]. This will help identify whether any single study disproportionately influences our overall findings and ensure that our conclusions are not unduly influenced by any single study, thereby enhancing the credibility of our synthesized evidence.

### Ethical Considerations

Ethical approval is not required for this protocol as it is a systematic review that includes secondary data from published studies. In this study, participants are not actively recruited, and data are not collected directly from them. The findings of the systematic review and meta-analysis will be disseminated through peer-reviewed publications. 

## Results

### Included Studies

**Figure 2:**
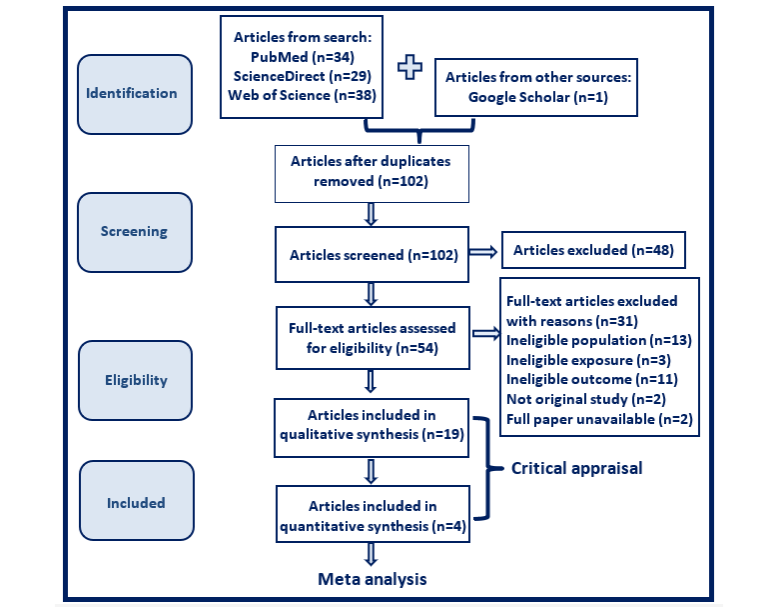
PRISMA (Preferred Reporting Items for Systematic Reviews and Meta-Analyses) flowchart.

A total of 19 qualitative and 4 quantitative studies were found relevant and were considered in our systematic review and meta-analysis (Figure 2). The qualitative studies were particularly valuable in guiding the methodology of our meta-analysis, informing our interpretation of the results, and shaping our conclusions. Additionally, they played a crucial role in constructing the literature review and establishing the background for our hypothesis by highlighting existing gaps in the literature. Of the 4 included quantitative studies, 3 are retrospective cohort studies and 1 is an observational, retrospective, case-control study. As for study group size, 1 cohort study was conducted on a population of less than 1000 participants [26], 2 studies (1 cohort [27] and 1 case-control [28]) involved more than 10,000 participants, and 1 cohort study included more than 6 million participants [29].

### Synthesis of Results

Our hypothesis being that overall centenarian mortality during COVID-19 is insignificant, we anticipate our synthesis of results from the meta-analysis pooled odds ratio and risk difference will reveal and support our hypothesis. We will also synthesize further evidence by running several subgroup analyses considering other variables in an attempt to reveal whether any differences in our outcome exist. For example, differences in outcome might be due to geographic location, age group, or type of residence.

## Discussion

### Interpretation

While extensive literature examines COVID-19 mortality in older adults [30,31], no systematic review has comprehensively synthesized global evidence specific to centenarians, a population with distinct biological resilience and vulnerability profiles. This study bridges this critical gap through a meta-analysis of mortality rates in SARS-CoV-2–positive centenarians and other older adults (≥60 years) across different care settings (long-term care facilities, hospitals, and home-based care). Using multiple estimators (raw-, logit-, and arcsine-transformed proportions; risk differences; and odds ratios) to quantify differential outcomes, our findings will help inform public health policies for older populations, integrate risk-stratified treatment protocols during future pandemics, and disseminate gerontological frameworks for protecting centenarian cohorts.

### Strengths Compared With Prior Work

Our study is different from the meta-analysis studies published regarding mortality in centenarians and other older adults. For example, a study highlighted that older individuals with dementia diagnosed with COVID-19 face a higher risk of mortality compared to those without dementia [32]. Another study found that, overall, frailty among older adults was linked to higher rates of COVID-19–related mortality compared with less frail counterparts [33]. A third study concluded that comorbidities contribute to increased COVID-19 mortality among older adults; however, this study relied on a single database [34]. Unlike the previous studies, our study looks at centenarian patients diagnosed with COVID-19 during the period from December 2019 to December 2024 and investigates their rate of mortality due to COVID-19 illness and not due to other or combined variables.

### Limitations, Implications for Practice, and Future Research

A key limitation was our narrow focus on mortality rates, omitting assessment of contributing factors—such as comorbidities, care settings, socioeconomic status, or health care access—which were rarely reported in the predominantly retrospective studies. Scant centenarian-specific data on these variables further precluded meaningful subgroup analyses. Future studies should adopt prospective designs with standardized risk factor assessments to identify predictors and predisposing factors of severe COVID-19.

Future studies should prioritize larger, more representative samples for this growing demographic to ensure adequate statistical power for complex analyses, including multivariable regression modeling of independent risk factors, confounder-adjusted effect estimation, and clinically meaningful subgroup stratification.

Future research should prioritize multicenter and international collaborations to assemble larger, more diverse cohorts of centenarians. This approach will enable pooled databases with enhanced statistical power, geographically representative sampling, and robust assessment of context-specific risk factors.

To address data sparsity and retrospective biases, we recommend prospective longitudinal cohorts capturing real-time clinical and social variables and stratified data collection disaggregating centenarians by age strata (eg, 100-105 years, 106-109 years, ≥110 years) and gender-specific responses for discovering mechanisms of resilience observed in some centenarians. International consortia (eg, Istituto di Ricovero e Cura a Carattere Scientifico-Istituto Nazionale di Ricovero e Cura per Anziani [IRCCS INRCA]) could operationalize this while ensuring standardization of data collection, definitions for symptoms, comorbidities, treatments, and outcomes, which maintain adequate power for minimizing risk of bias, data collection inconsistency, and imprecision.

### Conclusion

Considering centenarians, there is a notable absence of systematic reviews or meta-analysis studies consolidating knowledge about individuals in this demographic who succumbed to SARS-CoV-2 infection, even when considering available global data. Given the consensus that the centenarian population is a continuously growing demographic and is the most vulnerable to serious outcomes and consequences during pandemics, addressing this research gap is crucial for the informed development of public policies enabling countries to minimize the impacts on this population, particularly during health crises such as the COVID-19 pandemic. Filling this gap will contribute valuable insights to the field of gerontology and public health.

This meta-analysis provides the first comprehensive synthesis of COVID-19 mortality in centenarians, demonstrating that while rates are elevated, the difference from other older adults is modest and often not statistically significant. These findings challenge assumptions of inevitable poor outcomes in the oldest population and support the development of nuanced, evidence-based care frameworks for such populations.

## Appendix

Multimedia Appendix 1 COVID-19 protocol supplementary file.

## Abbreviations

PICOS: population, intervention, comparison, outcomes, and study
PRISMA: Preferred Reporting Items for Systematic Reviews and Meta-Analyses
SARS-CoV-2: severe acute respiratory syndrome coronavirus-2

## Data Availability

Data sharing is not available for this study yet as no data sets or analyses were generated at the time of submitting this protocol.
