# Supplementary material for: Centenarian Mortality Rate During COVID-19: Protocol for a Systematic Review and Meta-Analysis
Source: JMIR Res Protoc. 2025 Aug 13;14:e74068. doi: 10.2196/74068 (PMC12391843; doi:10.2196/74068)
Supplement: Multimedia Appendix 1 [file resprot_v14i1e74068_app1.docx]

# Centenarian Mortality Rate during COVID-19: Protocol for Systematic Review and Meta-Analysis

Shaima Ibrahim^a^, Wafa Abuelkheir Mataria^b^, Omnia Abdulrahim^b^, Sungsoo Chun^c^*

Institute of Global Health and Human Ecology, The American University in Cairo, New Cairo 11835, Egypt

^a^ Shaima Ibrahim, [sh_elamrousy@aucegypt.edu](mailto:sh_elamrousy@aucegypt.edu)

^b^ Wafa Abuelkheir Mataria, [wafamataria@aucegypt.edu](mailto:wafamataria@aucegypt.edu)

^b^ Omnia Abdulrahim, [omniaraheem@aucegypt.edu](mailto:omniaraheem@aucegypt.edu)

^c^ Institute of Global Health and Human Ecology, The American University in Cairo, Office # 2118, AUC Avenue, P.O. Box 74, New Cairo 11835, Egypt. *E-mail address: sungsoo.chun@aucegypt.edu (S. Chun).

**Corresponding Author*

The authors attest that there was no use of generative Artificial Intelligence (AI) technology in the generation of text, figures or other informational content of this manuscript.

| **Author, Pub Yr** | **Study** | **Region** | **Centenarian (Case)** | | | **Non-Centenarian (Control)** | | |
| --- | --- | --- | --- | --- | --- | --- | --- | --- |
|  |  |  | **Case** | **Case Death** | **Mortality %** | **Control** | **Control Death** | **Mortality %** |
| Couderc et al, 2021 [^[[1]](#endnote-1)^] | Retro-spective, cohort | 15 nursing homes in Marseille, France | 12 | 6 | 50.00 | 309 | 66 | 21.36 |
| Gallert et al., 2022 [^[[2]](#endnote-2)^] | Retro-spective, cohort | LTCF, Germany | 8,264 | 40 | 0.48 | 403,725 | 5,493 | 1.36 |
| Claudia et al., 2024 [^[[3]](#endnote-3)^] | Population based, cohort | Columbia | 1,005 | 373 | 37.11 | 6,312,867 | 3,508,691 | 55.58 |
| Cruces et al., 2024 [^[[4]](#endnote-4)^] | Population based, case-control | Basques country, Spain | 325 | 95 | 29.23 | 21,170 | 4,977 | 23.51 |

**Table S1: Details of Studies Meeting Inclusion Criteria.**

|  | **Table S2. Critical Appraisal questions for case control studies** | | | | | | | | | | |  |
| --- | --- | --- | --- | --- | --- | --- | --- | --- | --- | --- | --- | --- |
|  | Study | Q1 | Q2 | Q3 | Q4 | Q5 | Q6 | Q7 | Q8 | Q9 | Q10 |  |
| 1 | Cruses et al., 2024 |  |  |  |  |  |  |  |  |  |  |  |
|  |  |  |  |  |  |  |  |  |  |  |  |  |
|  |  | | | | | |  |  |  |  |  |  |
| Q1 | Were the groups comparable other than the presence of disease in cases or the absence of disease in controls? | | | | | | | | | | | |
| Q2 | Were cases and controls matched appropriately? | | | | | |  |  |  |  |  |  |
| Q3 | Were the same criteria used for identification of cases and controls? | | | | | | | | | Key |  |  |
| Q4 | Was exposure measured in a standard, valid and reliable way? | | | | | | | |  | Yes |  |  |
| Q5 | Was exposure measured in the same way for cases and controls? | | | | | | | |  | No |  |  |
| Q6 | Were confounding factors identified? | | | |  |  |  |  |  | NA |  |  |
| Q7 | Were strategies to deal with confounding factors stated? | | | | | | |  |  |  |  |  |
| Q8 | Were outcomes assessed in a standard, valid and reliable way for cases and controls? | | | | | | | | | | | |
| Q9 | Was the exposure period of interest long enough to be meaningful? | | | | | | | |  |  |  |  |
| Q10 | Was appropriate statistical analysis used? | | | | |  |  |  |  |  |  |  |

|  | **Table S3. Critical Appraisal questions for cohort studies.** | | | | | | | | | | | |
| --- | --- | --- | --- | --- | --- | --- | --- | --- | --- | --- | --- | --- |
|  | Study | Q1 | Q2 | Q3 | Q4 | Q5 | Q6 | Q7 | Q8 | Q9 | Q10 | Q11 |
| 1 | Couderc et al., 2021 |  |  |  |  |  |  |  |  |  |  |  |
| 2 | Gellert et al., 2022 |  |  |  |  |  |  |  |  |  |  |  |
| 3 | Claudia et al, 2024 |  |  |  |  |  |  |  |  |  |  |  |
|  |  |  |  |  |  |  |  |  |  |  |  |  |
|  |  | | | | |  |  |  |  |  |  |  |
| Q1 | Were the two groups similar and recruited from the same population? | | | | | | | | |  |  |  |
| Q2 | Were the exposures measured similarly to assign people to both exposed and unexposed groups? | | | | | | | | | | | |
| Q3 | Was the exposure measured in a valid and reliable way? | | | | | | |  |  |  |  |  |
| Q4 | Were confounding factors identified? | | | |  |  |  |  |  | Key |  |  |
| Q5 | Were strategies to deal with confounding factors stated? | | | | | | |  |  | Yes |  |  |
| Q6 | Were the groups/participants free of the outcome at the start of the study? | | | | | | | | | No |  |  |
| Q7 | Were the outcomes measured in a valid and reliable way? | | | | | | |  |  | NA |  |  |
| Q8 | Was the follow up time reported and sufficient to be long enough for outcomes to occur? | | | | | | | | | | | |
| Q9 | Was follow up complete, if not, were the reasons to loss to follow up described and explored? | | | | | | | | | | | |
| Q10 | Were strategies to address incomplete follow up utilized? | | | | | | |  |  |  |  |  |
| Q11 | Was appropriate statistical analysis used? | | | | |  |  |  |  |  |  |  |

1. Couderc et al., (2021). Centenarians in nursing homes during the COVID-19 pandemic. *Aging*, *13*(5), 6247–6257. <https://doi.org/10.18632/aging.202743> [↑](#endnote-ref-1)
2. Gellert et al, (2022). Centenarians From Long-Term Care Facilities and COVID-19–Relevant Hospital Admissions. *Journal of the American Medical Directors Association*, *23*(7), 1117–1118. <https://doi.org/10.1016/j.jamda.2022.05.009> [↑](#endnote-ref-2)
3. Birchenall-Jiménez et al., (2024). Centenarians in Colombia: A population-based cohort study on the impact of COVID 19. *Infectio*, 228–234. <https://doi.org/10.22354/24223794.1200> [↑](#endnote-ref-3)
4. Cruces et al., (2024). Analysis of response of centenarians of the Basque Country to COVID-19. *Journal of the American Geriatrics Society*, *72*(6), 1926–1929. <https://doi.org/10.1111/jgs.18853> [↑](#endnote-ref-4)
